# Multicolor Super-Resolution Microscopy of Protein Corona on Single Nanoparticles

**DOI:** 10.1021/acsami.2c06975

**Published:** 2022-08-12

**Authors:** Yuyang Wang, Paul E. D. Soto Rodriguez, Laura Woythe, Samuel Sánchez, Josep Samitier, Peter Zijlstra, Lorenzo Albertazzi

**Affiliations:** † Department of Applied Physics and Institute for Complex Molecular Systems (ICMS), Eindhoven University of Technology, 5612AZ Eindhoven, The Netherlands; ‡ Institute for Bioengineering of Catalonia (IBEC), The Barcelona Institute of Science and Technology, 08028 Barcelona, Spain; § Department of Biomedical Engineering and Institute for Complex Molecular Systems (ICMS), Eindhoven University of Technology, 5612AZ Eindhoven, The Netherlands; ∥ Institució Catalana de Recerca i Estudis Avançats (ICREA), Passeige Lluís Companys 23, 08010 Barcelona, Spain; ⊥ Department of Electronics and Biomedical Engineering, University of Barcelona (UB), 08028 Barcelona, Spain; # Biomedical Research Networking Center in Bioengineering, Biomaterials, and Nanomedicine (CIBER-BBN), 28029 Madrid, Spain

**Keywords:** STED microscopy, nanoparticles, protein corona, quantification, multicolor microscopy

## Abstract

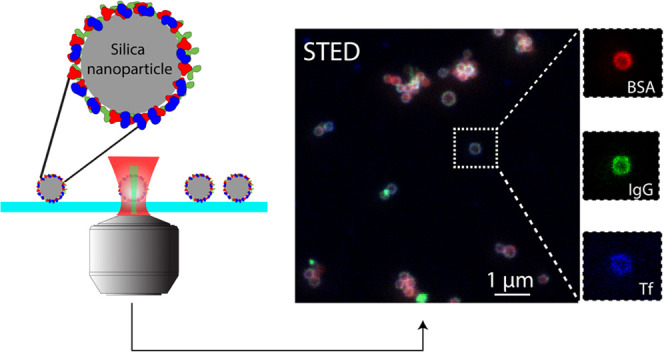

Nanoparticles represent a promising class of material for nanomedicine and molecular biosensing. The formation of a protein corona due to nonspecific particle–protein interactions is a determining factor for the biological fate of nanoparticles in vivo and strongly impacts the performance of nanoparticles when used as biosensors. Nonspecific interactions are usually highly heterogeneous, yet little is known about the heterogeneity of the protein corona that may lead to inter- and intraparticle differences in composition and protein distribution. Here, we present a super-resolution microscopic approach to study the protein corona on single silica nanoparticles and subsequent cellular interactions using multicolor stimulated emission depletion (STED) microscopy. We demonstrate that STED resolves structural features of protein corona on single particles including the distribution on the particle surface and the degree of protein internalization in porous particles. Using multicolor measurements of multiple labeled protein species, we determine the composition of the protein corona at the single-particle level. We quantify particle-to-particle differences in the composition and find that the composition is considerably influenced by the particle geometry. In a subsequent cellular uptake measurement, we demonstrate multicolor STED of protein corona on single particles internalized by cells. Our study shows that STED microscopy opens the window toward mechanistic understanding of protein coronas and aids in the rational design of nanoparticles as nanomedicines and biosensors.

## Introduction

The protein corona, or biomolecular corona, is a coat assembled on the surface of nanoparticles once they are administered into a biological medium containing biomolecules such as enzymes, proteins, or peptides.^1^ Upon being coated by protein corona, the nanoparticle’s biological fate will be largely determined by the protein coat rather than the underlying functionalized nanoparticle, giving a new identity that governs the cellular uptake process of the nanoparticle.^2,3^ Protein corona also impacts the performance of nanoparticles when used as label-free biosensors because the accessibility of surface functional groups is modulated.^4^ It is now known that the formation of protein corona is highly dynamic and dependent on a wide range of nanoparticle properties such as size,^5^ shape, and surface chemistry.^6−9^ Although significant progress in ensemble characterization of protein corona has been made, a deep and comprehensive understanding of protein–nanoparticle interaction at the molecular scale is vital for the development and rational design of particle-based nanomedicine and biosensors.^10^

The characterization of protein corona both in vitro and in vivo constitutes the essential work of protein corona studies, and a wide variety of analytical techniques have been used.^11,12^ Ensemble methods such as surface plasmon resonance,^1,5^ dynamic light scattering, ζ-potential,^13^ UV–vis spectroscopy, and fluorescence correlation spectroscopy^14,15^ have been used to monitor protein binding based on changes in particle size or surface charge. Gel electrophoresis,^16,17^ mass spectroscopy,^5,6,18^ and immunogold labeling^18^ have been used to identify and quantify the average amount of adsorbed protein on all particles. To achieve single-particle resolution, traditional microscopic techniques like atomic force microscopy,^19^ scanning electron microscopy,^17^ and transmission electron microscopy^13,16,20^ have been used to visualize protein corona on nanoparticles. However, sensitive, quantitative, noninvasive, and high-throughput techniques able to quantify the presence of multiple protein species at the single-particle level are desired, which should reveal a comprehensive picture of the protein corona.^10^

Fluorescence microscopy is a powerful complementary technique to overcome several limits in protein corona studies: it allows for biological-friendly sample preparation and high spatiotemporal resolution at single-particle level. Super-resolution microscopy (SRM) as a state-of-the-art technique has been rising as an indispensable tool to study various biological and synthetic materials.^21,22^ SRM can provide new insight into protein corona studies due to its ability to directly visualize protein corona on single particles with a spatial resolution that is not anymore limited by the diffraction limit (ca. 200–300 nm). Stochastic optical reconstruction microscopy (STORM) has been used to reveal protein corona on individual nanoparticles with a spatial resolution of <50 nm.^23,24^ STORM is however limited in throughput and temporal resolution since it heavily relies on posterior reconstruction of the image from single-molecule localizations detected over a long period of time for a single field of view, and in multicolor imaging limited by dye photophysics. Volume imaging is also challenging for STORM since it is normally based on total internal reflection excitation that provides little penetration depth into the sample.

Stimulated emission depletion (STED) microscopy in contrast is an SRM technique that is a powerful alternative to localization-based techniques.^25,26^ Based on confocal scanning of an engineered excitation spot, STED generates three-dimensional (3D) super-resolved images without sacrificing spatial resolution.^25,27^ With a resolution of 50–100 nm in both lateral and axial dimensions, STED has significantly contributed to the understanding of intracellular structures.^28−30^ STED microscopy is therefore a promising approach to study protein corona at the level of single nanoparticles where the multicolor, high-throughput, and high-resolution capabilities of STED open the window to not only investigate the composition of protein corona but also reveal particle-to-particle differences both in vitro and in cells.

Here, we demonstrate an approach to perform multicolor quantitative STED imaging of protein corona on single nanoparticles and their interactions with cells. We focus on amine-functionalized silica particles with average sizes ranging from 200–400 nm with different shapes and surface roughness. We use three blood plasma proteins, bovine serum albumin (BSA), immunoglobulin (IgG), and transferrin (Tf) as model protein corona components and labeled them with three STED-compatible dyes for multicolor STED analysis. By calibrating the system, we determine the fraction of the different protein species in the corona and correlate this with the size of individual NPs. We show that surface roughness and porosity have an unexpectedly large impact on the composition of the corona. We also demonstrate multicolor STED of the protein corona on single particles internalized by cells. Our study shows that STED microscopy is a valuable tool to generate a mechanistic understanding of the protein corona and in the rational design of nanoparticles as nanomedicines and biosensors.

## Results and Discussion

**Figure 1:**
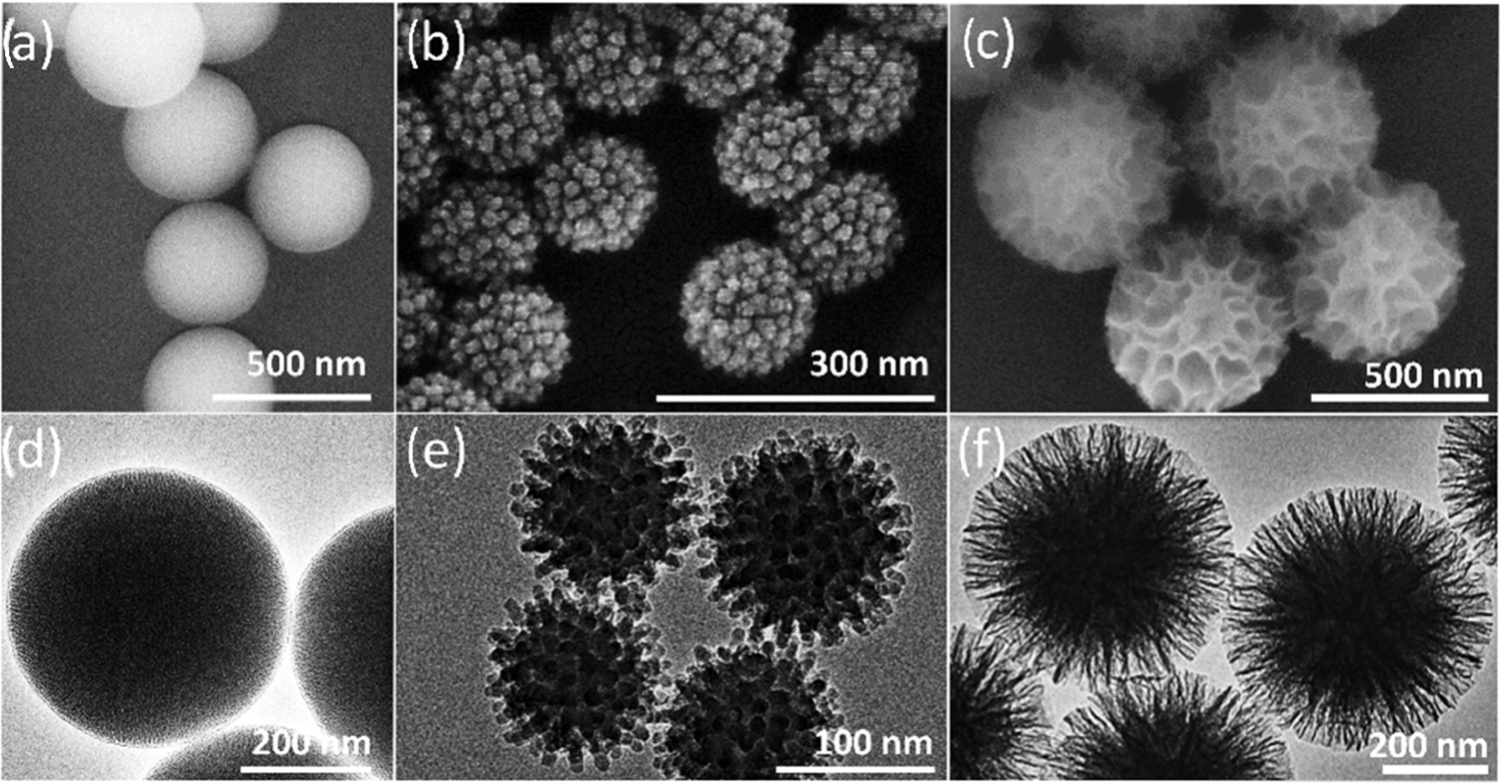
Morphological overview of silica nanoparticles for protein corona formation, displaying (a–c) SEM and (d–f) TEM images of MSN, VSN, and WSN nanoparticles, respectively.

Silica nanoparticles are versatile tools in biomedical applications and drug delivery due to their tunability of various properties by facile chemical synthesis.^31^ Many different types of mesoporous silica exist having different pore structures, particle sizes, and shapes.^32^ In this study, we have used three different types of synthesis approaches providing mesoporous silica nanoparticles (MSN) with three well-defined shapes. These nanoparticles were chosen due to their distinctly different surface roughness that could deliver an impact on protein corona formation. We employed a modified Stöber method to synthesize standard (MCM-41)-type MSN^33^ and two more recently described syntheses. Namely, through a novel single-micelle epitaxial growth approach in a low-concentration surfactant oil/water biphasic system, virus-like MSN (VSN) were obtained.^34^ The third synthesized particle is a KCC-1 type with fibrous pores^35^ and hereby named as wrinkled MSN (WSN). All three synthesis methods allow for precise control of the shape providing a well-defined surface configuration that enables the study of protein adsorption on different surfaces. The MSN has an average diameter of 400 nm as observed in electron microscope images, as shown in Figure 1a,d, whereas the VSN and WSN are sized between 200 and 300 nm, as shown in Figure 1b,f. Brunauer–Emmett–Teller (BET) adsorption measurements confirmed that WSN has a higher pore volume and smaller surface area than VSN (see Figure S4 in the Supporting Information for detailed BET results). Amino-functionalization was performed for the formation of protein corona, due to the high-efficiency adsorption of proteins to positively charged nanoparticle surfaces^23^ (see Figure S3 in the Supporting Information for ζ-potential results).

**Figure 2:**
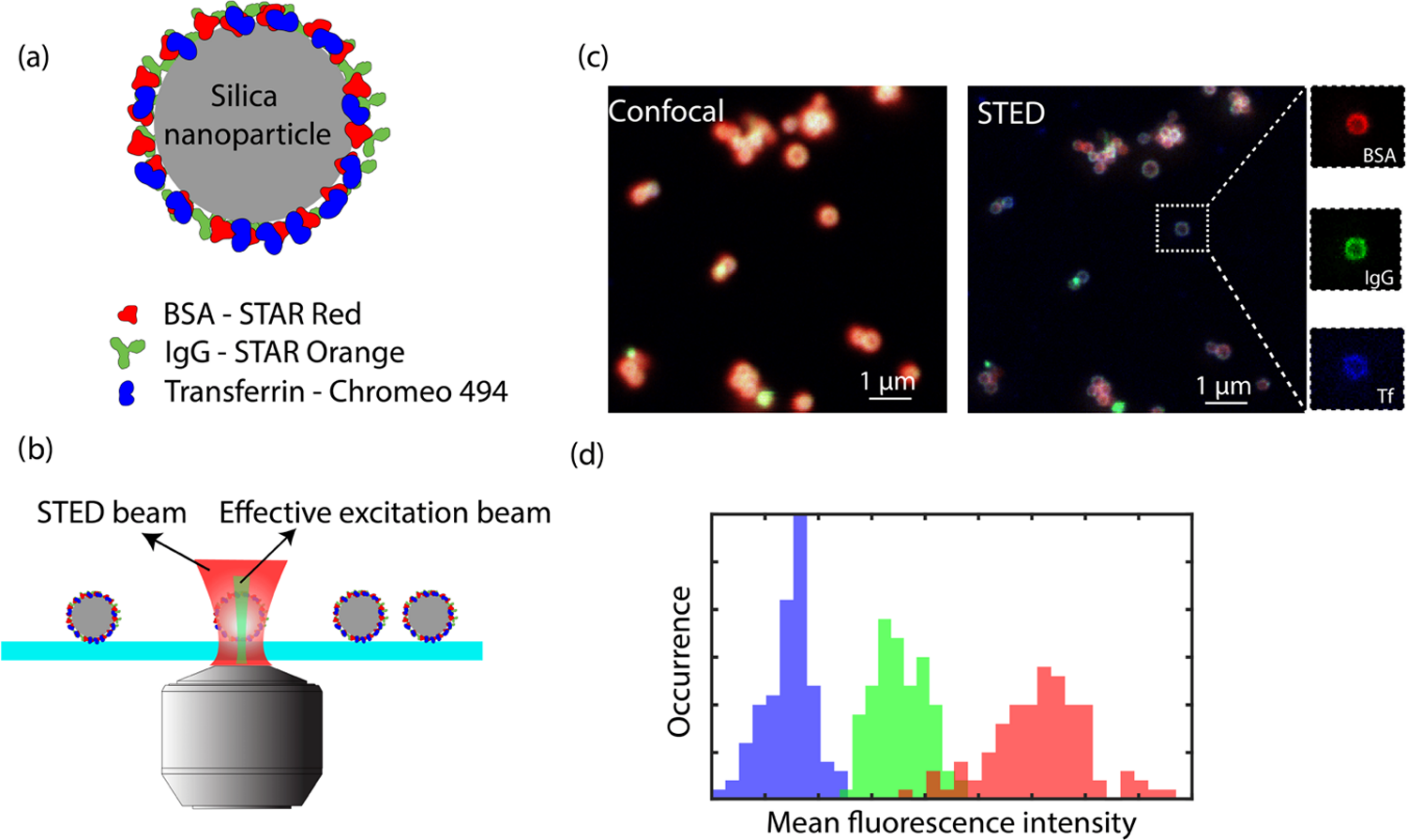
Workflow of STED microscopy of protein corona on nanoparticles. (a) Scheme of fluorescently labeled protein corona formation on a silica nanoparticle. (b) STED measurement of immobilized nanoparticle–protein corona complex. (c) Comparison of diffraction-limited confocal and super-resolution STED images of the same field of view, and zoom-in STED images of the same particles with BSA in red, IgG in green, and Tf in blue channels, respectively. (d) Illustrative histogram (not real data) showing the mean fluorescence intensities of single particles in different color channels with matching color codes as in (c).

Figure 2 demonstrates the workflow from sample preparation and STED image acquisition to data analysis. We first labeled MSN with the three most abundant blood plasma proteins BSA, IgG, and Tf (Figure 2a). BSA, IgG, and Tf were first labeled with STED-compatible Star Red, Star Orange, and Chromeo 494 dyes, respectively, via EDC/NHS chemistry. The three dyes were selected based on their optical properties, i.e., excitation/emission spectra and STED wavelength so that the three dyes can be sequentially measured and spectrally separated in different excitation and detection windows and yet the same STED depletion wavelength of 795 nm can be used to simplify the measurement. The degrees of labeling (DOLs) for all three proteins with respective dyes were determined by UV–vis spectroscopy, which were between 1 and 3 dyes per protein (see the Supporting Information). The labeling of nanoparticles with fluorescent proteins was further quantified by UV–vis spectroscopy (see Figure S1 in the Supporting Information). These properties were later used for the quantification of relative protein content on single particles.

The labeled nanoparticles were then immobilized on a clean glass coverslip with a photo-stabilizing imaging medium to ensure high STED imaging quality (Figure 2b). During STED imaging, different excitation lasers and detection windows were then sequentially activated to collect fluorescent signals from each dye. A comparison between the multicolor confocal and STED image of nanoparticles in the same field of view is shown in Figure 2a. Compared to the confocal image, STED not only resolved individual nanoparticles with higher resolution but also revealed the protein corona on single nanoparticles as a doughnut-shaped shell on the particle surface. This resolution enhancement is attributed to the STED mechanism, where a doughnut-shaped depletion beam suppresses fluorescence outside the center of the Gaussian excitation beam, achieving an effective excitation spot that is smaller than the diffraction limit. We observed this resolution enhancement in all three channels as shown in the breakup of a single-particle multicolor image in Figure 1c, verifying that the STED dye performance was sufficient to perform multicolor imaging of the corona. To quantify the relative content of proteins per particle, as shown in Figure 2c, the detected fluorescence intensities were corrected for the degree of labeling and the dye brightness. This allowed us to extract the fraction of each protein on a single particle. Imaging many particles across different fields of view then yielded histograms as sketched in Figure 2d and allowed us to analyze particle-to-particle differences.

**Figure 3:**
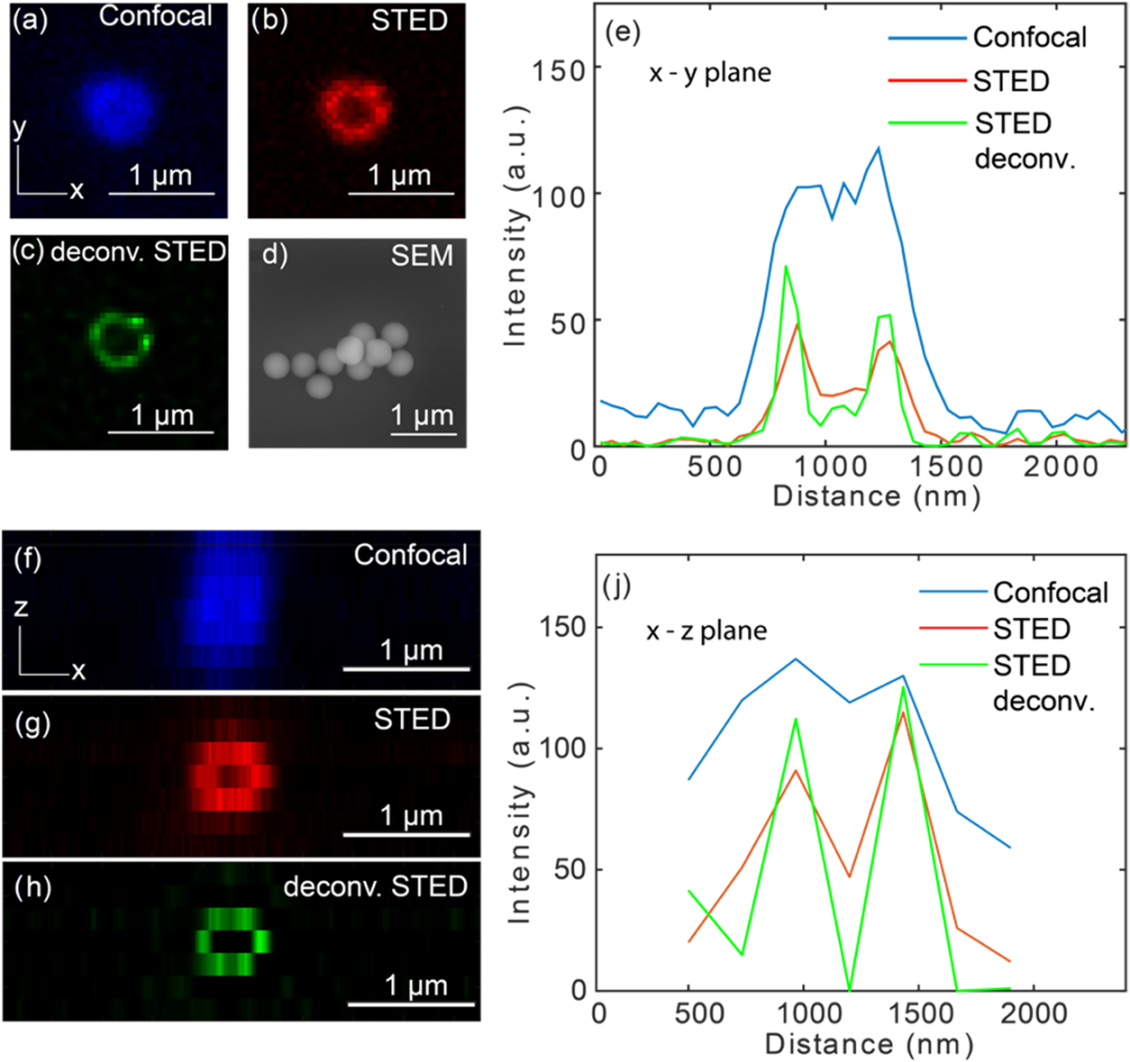
Comparison between confocal and STED imaging of MSN nanoparticles. (a–c) Microscope images scanned in the *x*–*y* plane (parallel to glass surface) for confocal, STED, and deconvoluted STED with a 75 nm PSF, respectively. The image is shown for a IgG-Star Orange coated silica nanoparticle with a smooth surface and diameter of ∼400 nm. (d) SEM image of the particles without a protein corona. (e) Line profile in the *x*–*y* plane of the same particle. (f–h) Microscope images scanned in the *x*–*z* plane for confocal, STED, and 75 nm ideal PSF deconvoluted STED respectively, and (j) line profile in the *x*–*z* plane of the same particle. In all microscope images, *x*–*y* scanning was performed with a pixel size of 20 nm, and *z* scanning with a pixel size of 200 nm.

In STED microscopy, the resolution enhancement depends on the intensity of the STED beam. By choosing the proper STED beam intensity, a compromise between resolution and photobleaching was found that enabled two-dimensional (2D) and 3D imaging of the particles. As shown in Figure 3c, the lateral (*x*–*y* plane) resolution increases drastically when performing single-color STED microscopy on silica nanoparticles that have an average diameter of ∼400 nm and smooth surface (Figure 3d). Further resolution improvement is observed when the STED images are deconvoluted with Gaussian-approximated ideal point spread function (PSF) with a full width at half-maximum (FWHM) of 75 nm. We found that STED successfully resolved protein corona structures on the surface of a nanoparticle, and deconvolution contributed to further enhancement of the contrast. The nonuniform intensity profile along the perimeter of a single particle reveals the heterogeneous distribution of protein corona on the nanoparticle surface induced likely by heterogeneous surface functionalization during protein adsorption, which was similarly reported in other works.^20,36^

To quantify the feature size after STED resolution enhancement, we show in Figure 3e the intensity profile along the center axis of a single nanoparticle’s confocal, STED, and deconvoluted STED image. In the confocal scan, no clear layer feature could be resolved due to the diffraction-limited resolution. We fitted the confocal line profile with a single Gaussian function and found an FWHM of 645 ± 36.1 nm, with a fitting uncertainty of 95% confidence interval. This measured width is attributed to the real diameter of the nanoparticle (∼400 nm) convoluted with a diffraction-limited confocal spot (∼230 for 640 nm laser focused with a NA 1.41 objective). In the STED scan, it was clearly observed that the protein corona’s layer structure was revealed, with two Gaussian-like peaks separated by the solid core of the nanoparticle. We fitted the raw and deconvolved STED image with a double Gaussian function and found minimal FWHMs of 219 ± 19.0 and 130 ± 13.2 nm, respectively. A diameter of 410 nm of the nanoparticle was determined by measuring the distance between fitted Gaussian peaks, matching well with the average diameter found in SEM (see the Supporting Information for more SEM data). The same measurement and analysis were performed for axial (*x*–*z* plane) confocal and STED scan, as shown in Figure 3f,j. We found that due to the lower axial confocal resolution, the protein corona could not be resolved, whereas STED accompanied by deconvolution significantly increased the resolution by a factor of ∼3. Quantification of the axial resolution proved challenging because sampling the particle at the Nyquist rate required >10 planes to be imaged, which was prohibited by photobleaching of the dye.

We note here that the measured protein layer thickness of >100 nm is larger than the expected nanometric monolayer hard protein corona formation.^37,38^ Considering that the nanoparticles had undergone centrifugal separation from excess proteins after incubation, it is unlikely that the protein corona would form as thick a layer structure. We attribute the apparent bigger feature size here mainly to the STED resolution of 50–100 nm in our measurement, limited by STED depletion intensity maximally allowed by photobleaching. The main factor determining the apparent layer thickness as imaged by STED is the finite axial resolution that causes a large (∼100 nm) slice of the 3D particle to be projected onto a 2D image. For this reason, the exact thickness of the protein corona cannot be reliably determined for these small particles.

We first demonstrate here the multicolor analysis of STED image data with the aim to quantify the relative protein content at the single-particle level. From the images, we extracted the mean fluorescence intensities of single nanoparticles in each color channel. The origin of the varying level of intensities is the combination of the degree of labeling and the relative amount of protein on each nanoparticle, in combination with the different brightness for each dye. We used the extracted intensities, combined with calibration as described in the Supporting Information to estimate the relative protein content displayed on a single nanoparticle level.

**Figure 4:**
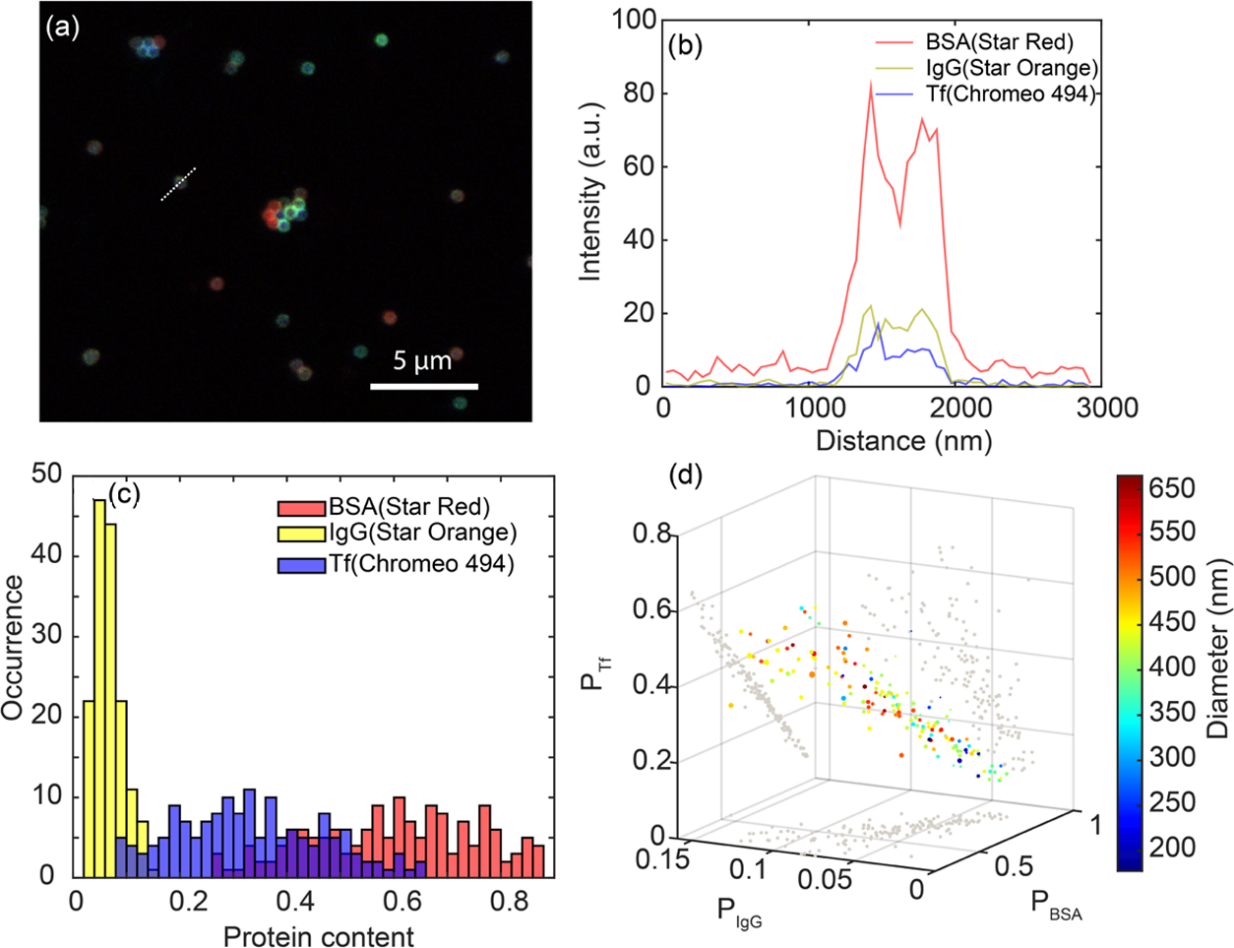
Multicolor STED imaging and quantification of MSN nanoparticles. (a) Three-color STED image of MSN nanoparticles immobilized on a coverslip. Pixel size in the image is 20 nm. In the image, BSA (Star Red) is shown in red, IgG (Star Orange) in green, and transferrin (Chromeo 494) in blue color scales that are adjusted for visibility. (b) Line profile in three-color channels across the single nanoparticle marked in (a). (c) Histogram of protein contents of all three proteins from protein corona coated on single MSN nanoparticles. (d) Scatter plot of protein content correlated with diameter (indicated by color scale) and pixel standard deviation (indicated by the size of scatter points).

In Figure 4a, we show the three-color STED image of 400 nm smooth silica nanoparticles (MSN) with amine functionality. It is clearly observed that fluorescent intensities collected from different color channels are vastly different on a single-particle level. From the line profile across a single particle in Figure 4b, we found that BSA with Star Red showed the highest intensity, while for IgG with Star Orange and Tf with Chromeo 494, a factor of 4 less intensity was detected from the same nanoparticle. Although less in intensity, these channels do not show a strongly reduced resolution due to the similar saturation intensity *I*_sat_ and the same STED depletion intensity *I* for all dyes.

We employed custom-made Matlab scripts (available in the Supporting Information) to perform the quantification of the relative protein content on single nanoparticles. The measured fluorescence intensity (sum of all pixel intensities on a single nanoparticle, corrected for background) from a single-color channel is proportional to the brightness of a single dye and the total number of fluorescent dyes in the excitation plane,i.e.,
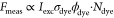
1where *I*_exc_ is the illumination power density, σ_dye_ is the absorption coefficient of the dye, ϕ_dye_ is the quantum yield of the dye, and *N*_dye_ is the number of dyes on a single particle. *N*_dye_ is then directly related to the number of proteins on a single particle via the degree of labeling (DOL)
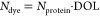
2

In contrast to single-color STED imaging, the quantitative comparison of signal intensity in multicolor images requires the correction of crosstalk between the different color channels. For this, we have performed crosstalk calibration measurements on nanoparticle samples labeled separately with only one kind of labeled proteins to determine a 3-by-3 crosstalk correction matrix σ_ct_, and the corrected fluorescence intensity
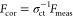
3

(see calculations of σ_ct_ in the Supporting Information). Therefore, by correcting measured fluorescence intensity with known values of σ_dye_ and ϕ_dye_, the amount of proteins can be semiquantitatively determined via
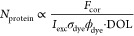
4

We have further ensured that excitation power density *I*_exc_ for all color channels remains the same during the measurement, we can then proceed to determine the ratiometric protein content by
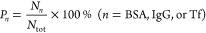
5where *N*_tot_ = *N*_BSA_ + *N*_IgG_ + *N*_Tf_ = 1 is the semiquantitative total amount of protein per particle.

In Figure 4c, we show a histogram calculated protein content *P*_*n*_ of all three protein species on single particles determined from a multicolor STED image. A total of ∼200 single nanoparticles were analyzed in a single scan across a sample area of 80 × 80 μm^2^, and from each single particle, the fluorescence intensities in all color channels corresponding to signals from three respective protein species were extracted for the calculation. All three protein species exhibit a monomodal and normal distribution with no clear appearance of multiple peaks or populations. For at least 70% of the single MSN, we find *P*_BSA_ > 50%, indicating that the corona on most particles is dominated by BSA, whereas IgG exhibited the lowest abundance. Below we will get back to this relative composition, where we find that the relative protein fraction strongly depends on the surface roughness of the particle.

We now turn our attention to the correlation between particle size and corona composition. STED imaging uniquely allows us to extract the size of each individual particle and then correlate the particle size to the corona composition. This analysis is shown in Figure 4d, where the relative protein content *P*_*n*_ is displayed in a 3D plot together with the particle diameter that is indicated by the color of the datapoints. The standard deviation of the fluorescence intensity on the periphery of each single particle is displayed as the size of the data point and indicates the uniformity of the protein corona coating. We observe a strong anticorrelation between the fraction of BSA and Tf, which is caused by the relatively low abundance of IgG. The corona being dominated by BSA and Tf, this automatically implies that a larger fraction of BSA is accompanied by a smaller fraction of Tf. No clear correlation was observed between particle diameter and the composition of the protein corona, likely because all particles are much larger than a protein and therefore essentially flat on nanometric length scales. We could not find any significant correlation in the number proteins beyond what is expected from a random sequential adsorption, indicating random adsorption of the proteins during corona formation. The relative abundance of the proteins we find is largely in agreement with previous STORM experiments, albeit transferrin was found to be the majority component there. This motivated us to investigate the effect of particle surface roughness as a possible parameter that dictates not only the amount of absorbed protein but also their relative abundance.

Apart from previously demonstrated MSN with a smooth surface and an average size of 400 nm, we synthesized virus-like silica nanoparticles (VSN) and wrinkled silica nanoparticles (WSN), both with average diameters between 200 and 300 nm. Although nanoparticles of similar sizes have been used in previous studies, the effect of surface topography has not been investigated before.^23,24,39^ Recent ensemble-averaged experiments indicated that surface roughness affects protein corona formation on polymeric nanoparticles and subsequent cellular uptake.^40^ We characterized here inorganic nanoparticles with distinctly different surface roughness to investigate the impact on protein corona formation and cellular uptake at the single-particle level.

**Figure 5:**
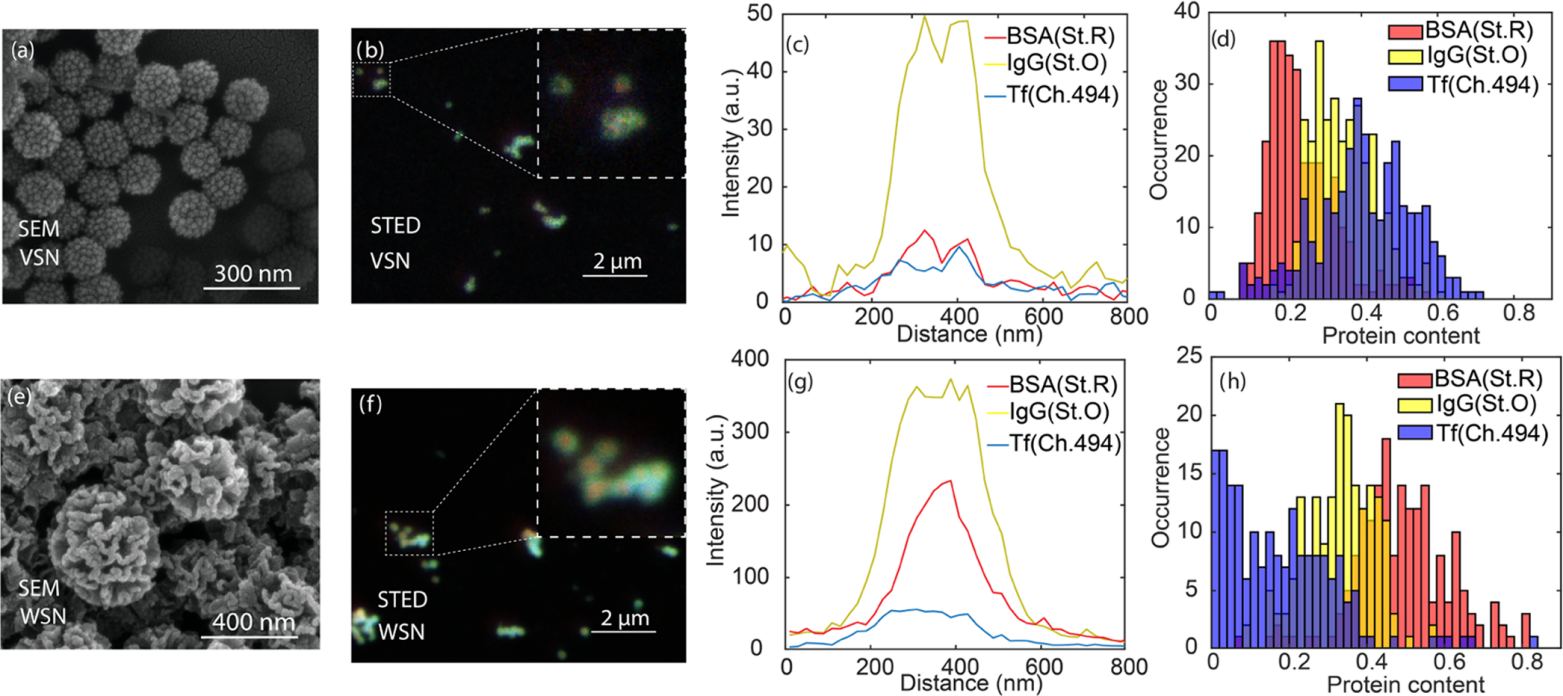
Roughness effect on PC formation. SEM images of (a) virus-like silica nanoparticles (VSN) and (e) wrinkled silica nanoparticles (WSN). Multicolor STED images and line profiles of coverslip-immobilized. (b, c) VSN and (f, g) WSN. STED images have a pixel size of 20 nm. (d, h) Histograms of protein contents of all three proteins from protein corona coated on single VSN and WSN nanoparticles.

In Figure 5a,e we show SEM images of VSN and WSN, respectively, with VSN showing spikes and WSN showing wrinkles extending to below the nanoparticle surface. Importantly, the surface features are nanometric and therefore occur on the same length scales as the size of the proteins. We performed multicolor STED microscopy with VSN and WSN nanoparticles labeled with all three fluorescent proteins, as shown in Figure 5b,f. As before, we observe strong particle-to-particle differences in the protein composition. For the VSN nanoparticles (Figure 5c), we find that the protein corona predominantly resides on the outer surface of the particle, which is evidenced by the two peaks and a central dip in the line profiles of BSA-Star Red and IgG-Star Orange.

Surprisingly, for the WSN particles, we find that the distinct surface features impact the corona formation by enabling smaller-sized proteins (particularly BSA) to migrate into the particle (Figure 5g). The BSA-Star Red line profiles exhibit a clear peak at the center of the particle, whereas IgG-Star Orange and Tf-Chromeo 494 exhibited a dip in the center of the line profile. This is indicative of BSA (the smallest of the three proteins) being more likely to migrate into the particle interior, whereas the larger IgG and Tf predominantly reside on the outer particle surface. In addition, we also found a higher overall number of proteins in the protein corona of WSN nanoparticles, which was revealed by an overall ∼10-fold higher fluorescence intensity in all three detection channels.

Surprisingly, we found that the relative protein contents are very different between smooth, VSN, and WSN nanoparticles. Figure 5d,h shows histograms of the relative protein contents on VSN and WSN nanoparticles, respectively. For VSN particles, Tf is the most abundant protein and BSA the least abundant, whereas for WSN particles, the reverse situation occurs. This is likely caused by the extra intake of BSA in the nanometric surface wrinkles of WSN nanoparticles, increasing their total BSA content relative to the other proteins. We note here the drastically different protein compositions in protein corona for MSN, VSN, and WSN nanoparticles, indicating that nanoparticle surface features can direct the protein corona composition depending on the size of the features relative to the size of the proteins.

Cellular uptake of nanoparticles is a fundamentally important process in drug delivery and is intrinsically a complicated process. Protein compositions and distinct protein species have been shown to strongly regulate the uptake of nanoparticles,^17,18,41^ as well as the shape and size of nanoparticles.^8,42,43^ Understanding the factors that determine the fate of nanoparticles is thus crucial for future success of rationally designed nanomedicine using nanoparticles as drug carriers.

**Figure 6:**
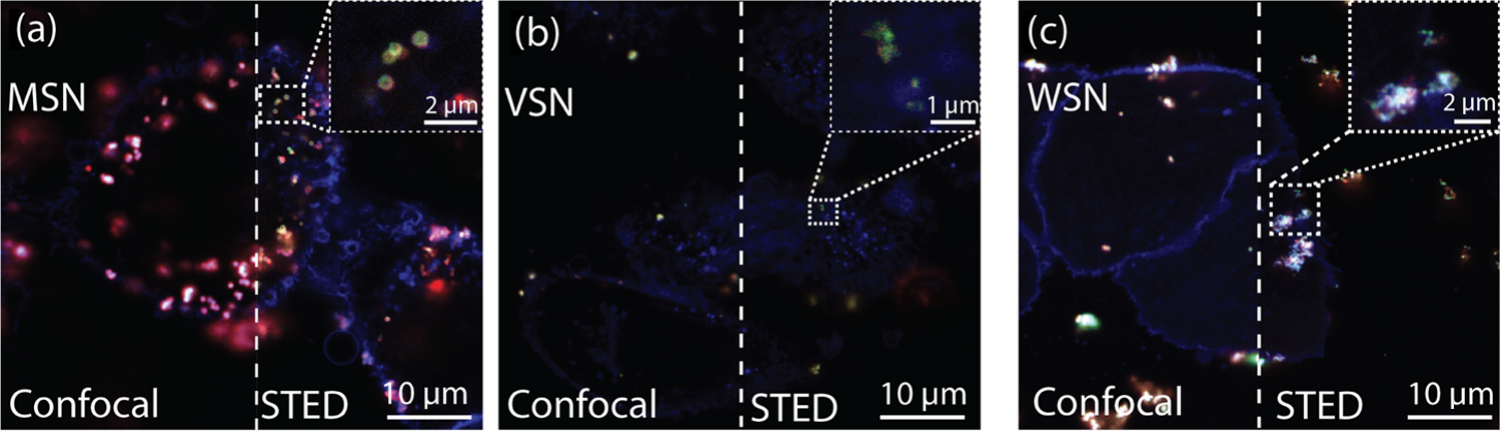
Interactions of protein corona-coated nanoparticles with MDA-MB-468 cells. Confocal and STED images of (a) MSN, (b) VSN, and (c) WSN nanoparticles after incubation with cells followed by fixation. Zoom-in STED images show nanoparticles internalized through cellular membranes. Pixel size in the images is 20 nm.

In this study, we investigated the cellular uptake of MSN, VSN, and WSN nanoparticles with multicolor STED microscopy as a proof of concept. We incubated protein corona-coated nanoparticles with MDA-MB-468 cells that were membrane-labeled with wheat germ agglutinin (WGA) and performed multicolor STED with WGA being measured in the same color channel as Chromeo 494 (transferrin label). Figure 6a,c shows the multicolor confocal and STED images of the three kinds of protein-coated nanoparticles after incubation with cells. All protein corona components on single nanoparticles of all three kinds internalized by cells with an average diameter of 200–400 nm could be clearly resolved by STED without significant changes in signal-to-background ratio (SBR), compared to STED measurements of immobilized nanoparticles as in Figures 4 and 5. The high SBR allows for direct localization of the nanoparticles in or out of cellular membranes, facilitating the study of internalized nanoparticles. We note here that due to lower overall number of proteins on VSN nanoparticles, the signals from Chromeo 494 channel are dominated by WGA during the same STED scan, particularly when nanoparticles are located close to labeled membrane, limiting the visualization of Chromeo 494-labeled transferrin.

The STED resolution of protein corona on single particles is not decreased in cellular measurement. In the line profiles of the multicolor STED images of single nanoparticles as shown in Figure S5, we find comparable FWHMs of protein corona layers on nanoparticles surface as those on immobilized nanoparticles, where VSN shows “BSA cores” and WSN with proteins residing in surface wrinkles. This also shows that the (hard) protein corona retains largely their presence and composition during cellular interaction of the nanoparticles.

We observed that different particles were internalized differently, with MSN and WSN nanoparticles being more likely to be internalized by cells, indicating likely a positive correlation of protein amount and extent of internalization. The similar protein content composition of MSN and WSN, both with BSA showing a dominating percentage, might have also played a role for the internalization. More studies need to be performed to isolate the effect of each protein species on cellular internalization of different nanoparticles. We find that significant aggregation happens during the progress of nanoparticle cell incubation for all nanoparticles, the mechanism of which can be revealed by a kinetics measurement using, e.g., STED measurement at different stages of cellular interactions. The higher extent of WSN nanoparticle aggregation may be correlated with the densely adsorbed proteins in the surface wrinkles, while this aggregation may also have happened in the cell culture medium.

## Conclusions

In summary, we studied protein corona composition on single nanoparticles with varying surface topography using multicolor STED. Correcting for crosstalk, degree of labeling, and optical properties of three dyes enabled the quantification of the relative amount of three proteins on the surface of each single particle. The improved resolution of STED allowed us to accurately determine whether the proteins were mainly present in the core and/or on the surface of the particles and enabled us to correlate the protein corona composition to the nanometric dimensions of each particle. We find large particle-to-particle differences in the protein corona composition within one sample, which we tentatively attribute to nanoscale variations in surface charge. We find that the relative amount of protein per particle does not strongly depend on particle size, but it is indeed influenced by the surface topography (nanoscale roughness) of the particle. In contrast to smooth and virus-like particles, the wrinkled nanoparticles are found to contain a large fraction of protein in their core. The nanometric resolution provided by STED showed surprisingly that one protein (BSA) diffuses into the core more efficiently likely due to its smaller size.

To investigate the consequence of different protein corona composition on cellular uptake, we also performed proof-of-concept measurements of protein corona composition inside cells. We have shown that multicolor STED imaging can be performed without loss of resolution inside fixated cells, paving the way to quantify the relationship between protein corona composition and cellular uptake. This quantification can be further performed by comparing protein corona compositions on internalized particles to particles that are not internalized. We have shown that multicolor STED reveals single nanoparticle heterogeneities of internalized protein corona-coated nanoparticles, and this is likely affected by the surface roughness features and the number of proteins on nanoparticle surfaces. Compared to previous single-molecule STORM measurement where images were acquired by the accumulative measurement of single-molecule events, our multicolor STED methodology provides an appealing alternative to directly image protein corona on single nanoparticles with higher-throughput, single-particle statistics and the ability to measure deeper in cells. As a future direction, we envision that multicolor STED measurement of single nanoparticles at different stages during the corona formation and cell internalization will reveal more mechanistic information.

## Methods

### Nanoparticle Synthesis

#### Synthesis of Mesoporous Silica (MSN)

For the synthesis, a modified Stöber method was used. For this method, tetraethyl orthosilicate (TEOS) was used as silica precursor, triethanolamine (TEOA) as a catalyst for the reaction, and hexadecyltrimethylammonium bromide (CTAB) as the porogenic agent. In a typical reaction, 570 mg of CTAB and 35 g of (TEOA) were dissolved in a flask containing 20 mL of DI water. The mixture was heated up in an oil bath to 95 °C. After 30 min, 1.5 mL of TEOS was added dropwise to the mixture. The reaction was left under magnetic stirring for a total of 2 h. The particles were collected and washed three times by centrifuging and dispersing in ethanol. To remove the porogenic agent, the particles were dispersed in a solution containing 30 mL of methanol and 1.8 mL of HCL and left in reflux for 24 h at 80 °C.

#### Synthesis of Wrinkled Silica (WSN)

For the synthesis, a modified method was used based on that reported by Bayal et al.^44^ For this method, CTAB (1 g), water (30 mL), and urea (600 mg) were mixed and a mixture of tetraethyl orthosilicate (TEOS) (6 mmol) and cyclohexane (30 mL) was dropwise added to form a lamellar phase of CTAB producing a wrinkled surface on silica. To stabilize the emulsion, 1-pentanol (1 mL) is dropwise added. The mixture was left for 16 h and refluxed at 70 °C under magnetic. The particles were collected and washed three times by centrifuging and dispersing in ethanol. To remove the CTAB, the samples were added to a solution of ammonium nitrate (160 mg) in ethanol (60 mL) at 60 °C for approximately 30 min; hereafter, the samples were washed with ethanol and dried in vacuum. Once dried, the samples were calcined overnight at 500 °C to remove any residual. The latter increases the amount of silanol groups on the surface having direct impact on the silica nanoparticle surface charge as it arises from their deprotonation.

#### Synthesis of Virus-like Silica (VSN)

The synthesis protocol follows a single-micelle epitaxial growth approach, which is favored in a low-surfactant-concentration oil/water biphase reaction system.^34^ A polar cationic surfactant, CTAB, served as the structural substrate. Tetraethyl orthosilicate (TEOS) was used as a precursor and NaOH as a catalyst for the reaction. The oil phase was provided by cyclohexane. In a typical synthesis, 1.0 g of the surfactant CTAB was added to 50 mL of deionized water containing 0.1 M NaOH and stirred for 2 h at 60 °C. A 20 mL mixture of TEOS in cyclohexane (20 v/v %) was added to the solution and kept at 60 °C with stirring for 2 weeks. The final solution was centrifuged at 12 K rpm for 5 min, washed with water (×3) and ethanol (×3), and sonicated for 15 min in between. To remove the CTAB templates, the samples were added to a solution of ammonium nitrate (160 mg) in ethanol (60 mL) at 60 °C for approximately 30 min; hereafter, the samples were washed with ethanol and dried in vacuum. Once dried, the samples were calcined overnight at 500 °C to remove any residual.

### Functionalization of VSNs (MSN/WSN) Using APTES (VSN-APTES)

The previously synthesized VSNs were dispersed in EtOH (2 mg/mL) by sonication (10 min). Once well dispersed, APTES (10 μL/mg of VSN) was added directly into the solution. The obtained mixture was stirred for 24 h at 50 °C with an end-to-end rotary shaker. Subsequently, the functionalized particles were centrifuged at 7.5 K rpm for 10 min and washed in ethanol (×3) and in water (×3), with sonication for 15 min between each centrifugation. Finally, aliquots (0.5 mL) were collected, centrifuged, and air-dried to determine the concentration of the VSNs suspension.

## Materials

Ethanol (EtOH, 99%), methanol (MeOH, 99%), hydrochloric acid (37% in water), ammonium nitrate, 99.999% metal basis (H_4_N_2_O_3_), ammonium hydroxide (25% in water), tetraethyl orthosilicate (TEOS, 99%), hexadecyltrimethylammonium bromide (CTAB, 99%), 3-aminopropyltriethoxysilane (APTES, 99%), glutaraldehyde (GA, 25% in water), urea (99.9%), phosphate-buffered saline (PBS), 1-pentanol (ACS reagent ⩾ 99,%), *N*,*N*-dimethylformamide (DMF) (for molecular biology ⩾ 99%), and succide anhydride were purchased from Thermo Fisher Scientific. Cyclohexane (ACS, 99 + %) was purchased from Cymit Química, S.L. MDA-MB-468 cells were purchased from ATCC (HTB-132). μ-Slide eight-well glass-bottom chambered coverslips (#1.5H) were obtained from Ibidi. DMEM (high glucose, no phenol red), penicillin-streptomycin, fetal bovine serum (qualified), Trypsin-EDTA (0.5%), WGA-488, and Nunc cell culture flasks were obtained from Thermo Fisher Scientific. Formaldehyde 37% was purchased from Sigma-Aldrich.

For transmission electron microscopy (TEM), bright-field images were taken with a JEOL JEM-2100 microscope. Scanning electron microscopy (SEM) images were obtained with an FEI NOVA NanoSEM 230 at 10 kV. The SEM samples were prepared by drop-casting 5 μL of particle solution (1 mg/mL) on a piece of a diced silicon wafer and left to dry. To increase the contrast, a few (ca. 2 nm) of metal (Pt) was sputtered on top for the dried particles with a LEICA EM ACE600 sputtering system. ζ-potential measurement was performed using a Wyatt Möbius coupled with an Atlas cell pressurization system.

### Protein Labeling and Corona Formation on Nanoparticles

Lyophilized powders of bovine serum albumin (BSA), immunoglobulin (IgG), and transferrin (Tf) from human serum were purchased from Sigma-Aldrich. All proteins were freshly dissolved at a concentration of 10 mg/mL (molar concentration 100–200 μM depending on molecular weights) in bicarbonate buffer (pH 9.2) prior to labeling. Commercial fluorescent dyes suitable for STED microscopy were purchased and directly used for protein labeling. Star Red NHS carbonate (638 nm/655 nm) and Star Orange NHS carbonate (589 nm/616 nm) were purchased from Abberior. Long Stokes shift dye Chromeo 494 (494 nm/628 nm) was purchased from Santa Cruz Biotechnology. All fluorescent dyes were dissolved in DMSO and kept at −20 °C until labeling.

For protein labeling, 5–10 μL of dye solution in DMSO was added to 250 μL of protein solution in bicarbonate buffer. Dye concentrations were controlled so that an excess of 2–3 times the final concentration was reached in the protein–dye mixture solution. The mixture was vortexed and kept at room temperature for 4 h. For multicolor measurement, a mixture of three proteins and a mixture of dye solutions were made at the same total molar concentration. After incubation, protein–dye mixture solution was dialyzed with Slide-A-lyzer Mini Dialysis Device (Thermo Fisher) and redispersed in HEPES buffer (pH 7.4). Chemical reagents to stop the NHS reaction were not used. The successful conjugation and the degree of labeling were confirmed and determined by UV–vis spectroscopy (results in the SI). For protein corona formation, as-labeled proteins were added to stock nanoparticle solutions (2 mg/mL) in HEPES buffer (pH 7.4) and kept on a shaking station for 4 h before being centrifuged twice at 14,000 rpm for 5 min and redispersed in the same HEPES buffer. The successful protein corona formation was confirmed by UV–vis spectroscopy (results in the SI).

### Cell Culture

MDA-MB-468 cells were cultured in high-glucose DMEM without phenol supplemented with 10% fetal bovine serum and penicillin-streptomycin (100U/ml) at 37 °C and 5% CO_2_. For incubation of cells with nanoparticles, the cells were detached from the culture flask using trypsin and seeded at a density of 50,000 cells/well in in Ibidi μ-slide eight-well glass-bottom chambered coverslips. The cells were incubated for 48 h at 37 °C and 5% CO_2_ before nanoparticle uptake experiments.

### STED Measurement

Prior to STED measurement, nanoparticles were either immobilized or incubated and formalin fixation. For immobilized samples, as-purified protein-coated nanoparticles were spin-coated on clean coverslips (0.17 mm), followed by gentle flushing with distilled water to rinse off salt stains. The coverslip was then covered with homemade Mowiol-glycerol mounting medium containing 1% DABCO for photostability.

For cell measurement, centrifuged nanoparticles were added to cell culture medium (DMEM, pH 7.4) at 20 times diluted concentration in an eight-well Ibidi glass-bottom coverslip at 200 μL/well. Incubation with MDA-MB-468 cells was done in a cell incubator at 37 °C for 2 h, followed by washing of cells with PBS and formaldehyde fixation (3,7%) for 10 min. After fixation, the cells were washed trice with PBS and stained with 0.5 μg/mL WGA-488 for 20 min. The sample was washed trice with PBS to remove excess WGA and then mounted directly on a STED microscope for measurement.

A commercial Expert Line setup (Abberior Instrument) was used for STED microscopy. All samples were imaged with a 100× NA 1.4 oil objective on an Olympus IX83 inverted microscope. Star Red labeled samples were excited with 640 nm, Star Orange with 561 nm, and Chromeo 494 with pulsed lasers (40 MHz). The power of excitation lasers ranged between 5 and 10 mW at the back aperture of the objective. To deplete the fluorescent signals from all three dyes, a pulsed STED beam of 795 nm at power ranging from 100 to 500 mW at back aperture applied depending on photophysics of different dyes was used. For all STED measurements, lateral and axial depletion was combined to realize approximately equal resolution in 3D. Deconvolution of the STED data was carried out using the deconvolution module in Abberior Imspector software. Estimated theoretical ideal PSF was generated, and Richardson–Lucy algorithm was used to perform deconvolution.

## Supporting Information Available

Degree of labeling determination, protein corona ensemble characterization, nanoparticle surface characterization, crosstalk correction, single-particle line profiles of protein corona internalized by cells, and Github link to download Matlab scripts for data analysis (Figures S1–S5) (PDF)

## References

[ref1] Lynch I.; Cedervall T.; Lundqvist M.; Cabaleiro-Lago C.; Linse S.; Dawson K. A. The Nanoparticle–Protein Complex as a Biological Entity; a Complex Fluids and Surface Science Challenge for the 21st Century. Adv. Colloid Interface Sci. 2007, 134–135, 167–174. 10.1016/j.cis.2007.04.021. 17574200

[ref2] Monopoli M. P.; Åberg C.; Salvati A.; Dawson K. A. Biomolecular Coronas Provide the Biological Identity of Nanosized Materials. Nat. Nanotechnol. 2012, 7, 779–786. 10.1038/nnano.2012.207. 23212421

[ref3] Monopoli M. P.; Walczyk D.; Campbell A.; Elia G.; Lynch I.; Baldelli Bombelli F.; Dawson K. A. Physical–Chemical Aspects of Protein Corona: Relevance to in Vitro and in Vivo Biological Impacts of Nanoparticles. J. Am. Chem. Soc. 2011, 133, 2525–2534. 10.1021/ja107583h. 21288025

[ref4] Beuwer M. A.; Prins M. W. J.; Zijlstra P. Stochastic Protein Interactions Monitored by Hundreds of Single-Molecule Plasmonic Biosensors. Nano Lett. 2015, 15, 3507–3511. 10.1021/acs.nanolett.5b00872. 25833294

[ref5] Cedervall T.; Lynch I.; Lindman S.; Berggard T.; Thulin E.; Nilsson H.; Dawson K. A.; Linse S. Understanding the Nanoparticle-Protein Corona Using Methods to Quantify Exchange Rates and Affinities of Proteins for Nanoparticles. Proc. Natl. Acad. Sci. U.S.A 2007, 104, 2050–2055. 10.1073/pnas.0608582104. 17267609 PMC1892985

[ref6] Lundqvist M.; Stigler J.; Elia G.; Lynch I.; Cedervall T.; Dawson K. A. Nanoparticle Size and Surface Properties Determine the Protein Corona with Possible Implications for Biological Impacts. Proc. Natl. Acad. Sci. U.S.A 2008, 105, 14265–14270. 10.1073/pnas.0805135105. 18809927 PMC2567179

[ref7] Lundqvist M.; Stigler J.; Cedervall T.; Berggård T.; Flanagan M. B.; Lynch I.; Elia G.; Dawson K. The Evolution of the Protein Corona around Nanoparticles: A Test Study. ACS Nano 2011, 5, 7503–7509. 10.1021/nn202458g. 21861491

[ref8] Visalakshan R. M.; García L. E. G.; Benzigar M. R.; Ghazaryan A.; Simon J.; Mierczynska-Vasilev A.; Michl T. D.; Vinu A.; Mailänder V.; Morsbach S.; Landfester K.; Vasilev K. The Influence of Nanoparticle Shape on Protein Corona Formation. Small 2020, 16, 2000285 10.1002/smll.202000285. 32406176

[ref9] Docter D.; Westmeier D.; Markiewicz M.; Stolte S.; Knauer S. K.; Stauber R. H. The Nanoparticle Biomolecule Corona: Lessons Learned – Challenge Accepted?. Chem. Soc. Rev. 2015, 44, 6094–6121. 10.1039/C5CS00217F. 26065524

[ref10] Nienhaus K.; Nienhaus G. U. Towards a Molecular-Level Understanding of the Protein Corona around Nanoparticles – Recent Advances and Persisting Challenges. Curr. Opin. Biomed. Eng. 2019, 10, 11–22. 10.1016/j.cobme.2019.01.002.

[ref11] Baimanov D.; Cai R.; Chen C. Understanding the Chemical Nature of Nanoparticle–Protein Interactions. Bioconjugate Chem. 2019, 30, 1923–1937. 10.1021/acs.bioconjchem.9b00348. 31259537

[ref12] Nienhaus K.; Wang H.; Nienhaus G. U. Nanoparticles for Biomedical Applications: Exploring and Exploiting Molecular Interactions at the Nano-Bio Interface. Mater. Today Adv. 2020, 5, 100036 10.1016/j.mtadv.2019.100036.

[ref13] Kokkinopoulou M.; Simon J.; Landfester K.; Mailänder V.; Lieberwirth I. Visualization of the Protein Corona: Towards a Biomolecular Understanding of Nanoparticle-Cell-Interactions. Nanoscale 2017, 9, 8858–8870. 10.1039/C7NR02977B. 28632260

[ref14] Shang L.; Nienhaus G. U. In Situ Characterization of Protein Adsorption onto Nanoparticles by Fluorescence Correlation Spectroscopy. Acc. Chem. Res. 2017, 50, 387–395. 10.1021/acs.accounts.6b00579. 28145686

[ref15] Röcker C.; Pötzl M.; Zhang F.; Parak W. J.; Nienhaus G. U. A Quantitative Fluorescence Study of Protein Monolayer Formation on Colloidal Nanoparticles. Nat. Nanotechnol. 2009, 4, 577–580. 10.1038/nnano.2009.195. 19734930

[ref16] Yin H.; Chen R.; Casey P. S.; Ke P. C.; Davis T. P.; Chen C. Reducing the Cytotoxicity of ZnO Nanoparticles by a Pre-Formed Protein Corona in a Supplemented Cell Culture Medium. RSC Adv. 2015, 5, 73963–73973. 10.1039/C5RA14870G.

[ref17] Mirshafiee V.; Kim R.; Park S.; Mahmoudi M.; Kraft M. L. Impact of Protein Pre-Coating on the Protein Corona Composition and Nanoparticle Cellular Uptake. Biomaterials 2016, 75, 295–304. 10.1016/j.biomaterials.2015.10.019. 26513421

[ref18] Tonigold M.; Simon J.; Estupiñán D.; Kokkinopoulou M.; Reinholz J.; Kintzel U.; Kaltbeitzel A.; Renz P.; Domogalla M. P.; Steinbrink K.; Lieberwirth I.; Crespy D.; Landfester K.; Mailänder V. Pre-Adsorption of Antibodies Enables Targeting of Nanocarriers despite a Biomolecular Corona. Nat. Nanotechnol. 2018, 13, 862–869. 10.1038/s41565-018-0171-6. 29915272

[ref19] Chong Y.; Ge C.; Yang Z.; Garate J. A.; Gu Z.; Weber J. K.; Liu J.; Zhou R. Reduced Cytotoxicity of Graphene Nanosheets Mediated by Blood-Protein Coating. ACS Nano 2015, 9, 5713–5724. 10.1021/nn5066606. 26040772

[ref20] Kokkinopoulou M.; Simon J.; Landfester K.; Mailänder V.; Lieberwirth I. Visualizing the Protein Corona: A Qualitative and Quantitative Approach towards the Nano-Bio-Interface. Microsc. Microanal. 2017, 23, 1188–1189. 10.1017/S1431927617006602.

[ref21] Pujals S.; Albertazzi L. Super-Resolution Microscopy for Nanomedicine Research. ACS Nano 2019, 13, 9707–9712. 10.1021/acsnano.9b05289. 31424198 PMC6764015

[ref22] Pujals S.; Feiner-Gracia N.; Delcanale P.; Voets I.; Albertazzi L. Super-Resolution Microscopy as a Powerful Tool to Study Complex Synthetic Materials. Nat. Rev. Chem. 2019, 3, 68–84. 10.1038/s41570-018-0070-2.

[ref23] Feiner-Gracia N.; Beck M.; Pujals S.; Tosi S.; Mandal T.; Buske C.; Linden M.; Albertazzi L. Super-Resolution Microscopy Unveils Dynamic Heterogeneities in Nanoparticle Protein Corona. Small 2017, 13, 1701631 10.1002/smll.201701631. 28922574

[ref24] Delcanale P.; Miret-Ontiveros B.; Arista-Romero M.; Pujals S.; Albertazzi L. Nanoscale Mapping Functional Sites on Nanoparticles by Points Accumulation for Imaging in Nanoscale Topography (PAINT). ACS Nano 2018, 12, 7629–7637. 10.1021/acsnano.7b09063. 30048592

[ref25] Hell S. W.; Wichmann J. Breaking the Diffraction Resolution Limit by Stimulated Emission: Stimulated-Emission-Depletion Fluorescence Microscopy. Opt. Lett. 1994, 19, 780–782. 10.1364/OL.19.000780. 19844443

[ref26] Hell S. W. Far-Field Optical Nanoscopy. Science 2007, 316, 1153–1158. 10.1126/science.1137395. 17525330

[ref27] Hell S. W.; Sahl S. J.; Bates M.; Zhuang X.; Heintzmann R.; Booth M. J.; Bewersdorf J.; Shtengel G.; Hess H.; Tinnefeld P.; Honigmann A.; Jakobs S.; Testa I.; Cognet L.; Lounis B.; Ewers H.; Davis S. J.; Eggeling C.; Klenerman D.; Willig K. I.; Vicidomini G.; Castello M.; Diaspro A.; Cordes T. The 2015 Super-Resolution Microscopy Roadmap. J. Phys. D: Appl. Phys. 2015, 48, 443001 10.1088/0022-3727/48/44/443001.

[ref28] Müller T.; Schumann C.; Kraegeloh A. STED Microscopy and Its Applications: New Insights into Cellular Processes on the Nanoscale. ChemPhysChem 2012, 13, 1986–2000. 10.1002/cphc.201100986. 22374829

[ref29] Barentine A. E. S.; Schroeder L. K.; Graff M.; Baddeley D.; Bewersdorf J. Simultaneously Measuring Image Features and Resolution in Live-Cell STED Images. Biophys. J. 2018, 115, 951–956. 10.1016/j.bpj.2018.07.028. 30139523 PMC6139878

[ref30] Heine J.; Wurm C. A.; Keller-Findeisen J.; Schönle A.; Harke B.; Reuss M.; Winter F. R.; Donnert G. Three Dimensional Live-Cell STED Microscopy at Increased Depth Using a Water Immersion Objective. Rev. Sci. Instrum. 2018, 89, 053701 10.1063/1.5020249. 29864829

[ref31] Janjua T. I.; Cao Y.; Yu C.; Popat A. Clinical Translation of Silica Nanoparticles. Nat. Rev. Mater. 2021, 6, 1072–1074. 10.1038/s41578-021-00385-x. 34642607 PMC8496429

[ref32] Narayan R.; Nayak U. Y.; Raichur A. M.; Garg S. Mesoporous Silica Nanoparticles: A Comprehensive Review on Synthesis and Recent Advances. Pharmaceutics 2018, 10, 118 10.3390/pharmaceutics10030118. PMC6160987 30082647

[ref33] Hortelão A. C.; Patiño T.; Perez-Jiménez A.; Blanco À.; Sánchez S. Enzyme-Powered Nanobots Enhance Anticancer Drug Delivery. Adv. Funct. Mater. 2018, 28, 1705086 10.1002/adfm.201705086.

[ref34] Wang W.; Wang P.; Tang X.; Elzatahry A. A.; Wang S.; Al-Dahyan D.; Zhao M.; Yao C.; Hung C.-T.; Zhu X.; Zhao T.; Li X.; Zhang F.; Zhao D. Facile Synthesis of Uniform Virus-like Mesoporous Silica Nanoparticles for Enhanced Cellular Internalization. ACS Cent. Sci. 2017, 3, 839–846. 10.1021/acscentsci.7b00257. 28852697 PMC5571464

[ref35] Maity A.; Belgamwar R.; Polshettiwar V. Facile Synthesis to Tune Size, Textural Properties and Fiber Density of Dendritic Fibrous Nanosilica for Applications in Catalysis and CO2 Capture. Nat. Protoc. 2019, 14, 2177–2204. 10.1038/s41596-019-0177-z. 31189974

[ref36] Post R. A. J.; van der Zwaag D.; Bet G.; Wijnands S. P. W.; Albertazzi L.; Meijer E. W.; van der Hofstad R. W. A Stochastic View on Surface Inhomogeneity of Nanoparticles. Nat. Commun. 2019, 10, 1663 10.1038/s41467-019-09595-y. 30971686 PMC6458121

[ref37] Marichal L.; Giraudon--Colas G.; Cousin F.; Thill A.; Labarre J.; Boulard Y.; Aude J.-C.; Pin S.; Renault J. P. Protein–Nanoparticle Interactions: What Are the Protein–Corona Thickness and Organization?. Langmuir 2019, 35, 10831–10837. 10.1021/acs.langmuir.9b01373. 31333024

[ref38] Kihara S.; Ghosh S.; McDougall D. R.; Whitten A. E.; Mata J. P.; Köper I.; McGillivray D. J. Structure of Soft and Hard Protein Corona around Polystyrene Nanoplastics—Particle Size and Protein Types. Biointerphases 2020, 15, 051002 10.1116/6.0000404. 32948094

[ref39] Monopoli M. P.; Åberg C.; Salvati A.; Dawson K. A. Biomolecular Coronas Provide the Biological Identity of Nanosized Materials. Nat. Nanotechnol. 2012, 7, 779–786. 10.1038/nnano.2012.207. 23212421

[ref40] Piloni A.; Ken Wong C.; Chen F.; Lord M.; Walther A.; H Stenzel M. Surface Roughness Influences the Protein Corona Formation of Glycosylated Nanoparticles and Alter Their Cellular Uptake. Nanoscale 2019, 11, 23259–23267. 10.1039/C9NR06835J. 31782458

[ref41] Ritz S.; Schöttler S.; Kotman N.; Baier G.; Musyanovych A.; Kuharev J.; Landfester K.; Schild H.; Jahn O.; Tenzer S.; Mailänder V. Protein Corona of Nanoparticles: Distinct Proteins Regulate the Cellular Uptake. Biomacromolecules 2015, 16, 1311–1321. 10.1021/acs.biomac.5b00108. 25794196

[ref42] Ding L.; Yao C.; Yin X.; Li C.; Huang Y.; Wu M.; Wang B.; Guo X.; Wang Y.; Wu M. Size, Shape, and Protein Corona Determine Cellular Uptake and Removal Mechanisms of Gold Nanoparticles. Small 2018, 14, 1801451 10.1002/smll.201801451. 30239120

[ref43] Sen Gupta A. Role of Particle Size, Shape, and Stiffness in Design of Intravascular Drug Delivery Systems: Insights from Computations, Experiments, and Nature. Wiley Interdiscip. Rev.: Nanomed. Nanobiotechnol. 2016, 8, 255–270. 10.1002/wnan.1362. 26306941

[ref44] Bayal N.; Singh B.; Singh R.; Polshettiwar V. Size and Fiber Density Controlled Synthesis of Fibrous Nanosilica Spheres (KCC-1). Sci. Rep. 2016, 6, 24888 10.1038/srep24888. 27118152 PMC4846819

