# Supplementary material for: Multicolor Super-Resolution Microscopy of Protein Corona on Single Nanoparticles
Source: ACS Appl Mater Interfaces. 2022 Aug 12;14(33):37345–55. doi: 10.1021/acsami.2c06975 (PMC9412947; doi:10.1021/acsami.2c06975)
Supplement: Supplementary file 1 — am2c06975_si_001.pdf [file am2c06975_si_001.pdf]

Supporting Information for:

Multicolor super-resolution microscopy of protein corona on single nanoparticles

Yuyang Wang[1], Paul E.D. Soto Rodriguez[2 †], Laura Woythe[3], Samuel Sánchez [2,4], Josep Samitier[2,5,6], Peter Zijlstra[1], Lorenzo Albertazzi[2,3*]*

[1]Department of Applied Physics and Institute for Complex Molecular Systems (ICMS), Eindhoven University of Technology, 5612AZ Eindhoven, The Netherlands

[2]Institute for Bioengineering of Catalonia (IBEC), The Barcelona Institute of Science and Technology, 08028 Barcelona, Spain

[3]Department of Biomedical Engineering and Institute for Complex Molecular Systems (ICMS), Eindhoven University of Technology, 5612AZ Eindhoven, The Netherlands

[4]Institució Catalana de Recerca i Estudis Avançats (ICREA), Pg. Lluís Companys 23, 08010 Barcelona, Spain

[5]Department of Electronics and Biomedical Engineering, University of Barcelona (UB), 08028 Barcelona, Spain

[6]Biomedical Research Networking Center in Bioengineering, Biomaterials, and Nanomedicine (CIBER-BBN), 28029 Madrid, Spain

Contents

Degree of labeling (DOL) measurement 3

Protein corona formation with fluorescently labeled proteins 5

Nanoparticle surface characterization.. 6

Cross-talk correction 7

Line profiles of protein corona on single nanoparticles internalized by cells.. 9

Matlab codes for STED analysis 10

Degree of labeling (DOL) measurement

We find the DOL of three proteins: BSA with Star Red, IgG with Star Orange, and Tf with Chromeo 494 by UV-Vis absorption measurement. UV-Vis spectra of proteins before and after labeling were measured. The DOL is defined by the moles of dye per mole protein $DOL = \frac{A_{max}}{\epsilon_{dye} \cdot c_{protein}}$, where protein concentration $c_{protein} = \frac{(A_{280} - (A_{max} \cdot CF))}{\epsilon\_protein}$, where A_{280} is the absorption of the solution at 280 nm contributed by both the protein and the dye, and A_{max} the maximum absorption from the dye at the maximum emission wavelength.

For DOL calculation, properties of the dyes are needed. We sourced photophysical properties of all dyes from manufacturers and used them for the calculations. In Table 1 we show the quantum yield, CF_{280} values and extinction coefficients for all three dyes. In Table 2 we show the extinction coefficients and molecular weights of all proteins.

Table 1 Photophysical properties of used fluorescent dyes

	STAR red	STAR orange	Chromeo 494
ϕ_{dye}	0.55	0.55	0.15
CF_{280}	0.32	0.56	0.11
ϵ_{dye}	120,000 $M^{-1}cm^{-1}$	95,000 $M^{-1}cm^{-1}$	55,000 $M^{-1}cm^{-1}$

Table 2 Extinction and molecular weights of used proteins

	BSA	IgG	Transferrin
ϵ_{molar}*	43,824 $M^{-1}cm^{-1}$	210,000 $M^{-1}cm^{-1}$	89,600 $M^{-1}cm^{-1}$
$\epsilon_{percent}$	6.6	14	11.2
Molecular weight	66,400	150,000	80,000

$$* \epsilon_{molar} \times 10 = \epsilon_{percent} \times molecular\ weght$$

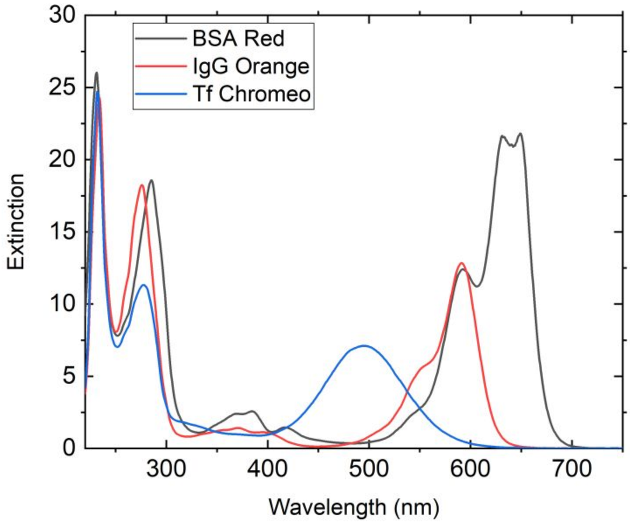

Figure S1 UV-Vis spectra of dye-labeled protein solutions

In Figure S1, we show the UV-Vis spectra of dye-labeled proteins, where protein absorptions at 280 nm, A_{280}, and dye absorption peaks A_{max} at respective peak wavelengths were clearly found. The measured A_280 and A_{max} and calculated protein concentration $c_{protein}$ and DOL are presented in Table 3.

Table 3 Calculated DOLs of protein labeling

	BSA Star Red	IgG Star Orange	Tf Chromeo 494
A_{280} (10 mm)	18.6	18.2	11.3
A_{max} (10 mm)	21.6	12.8	7.10
Calculated $c_{protein}$	266 uM	52 uM	131 uM
Calculated DOL	0.67	2.5	0.99

Protein corona formation with fluorescently labeled proteins

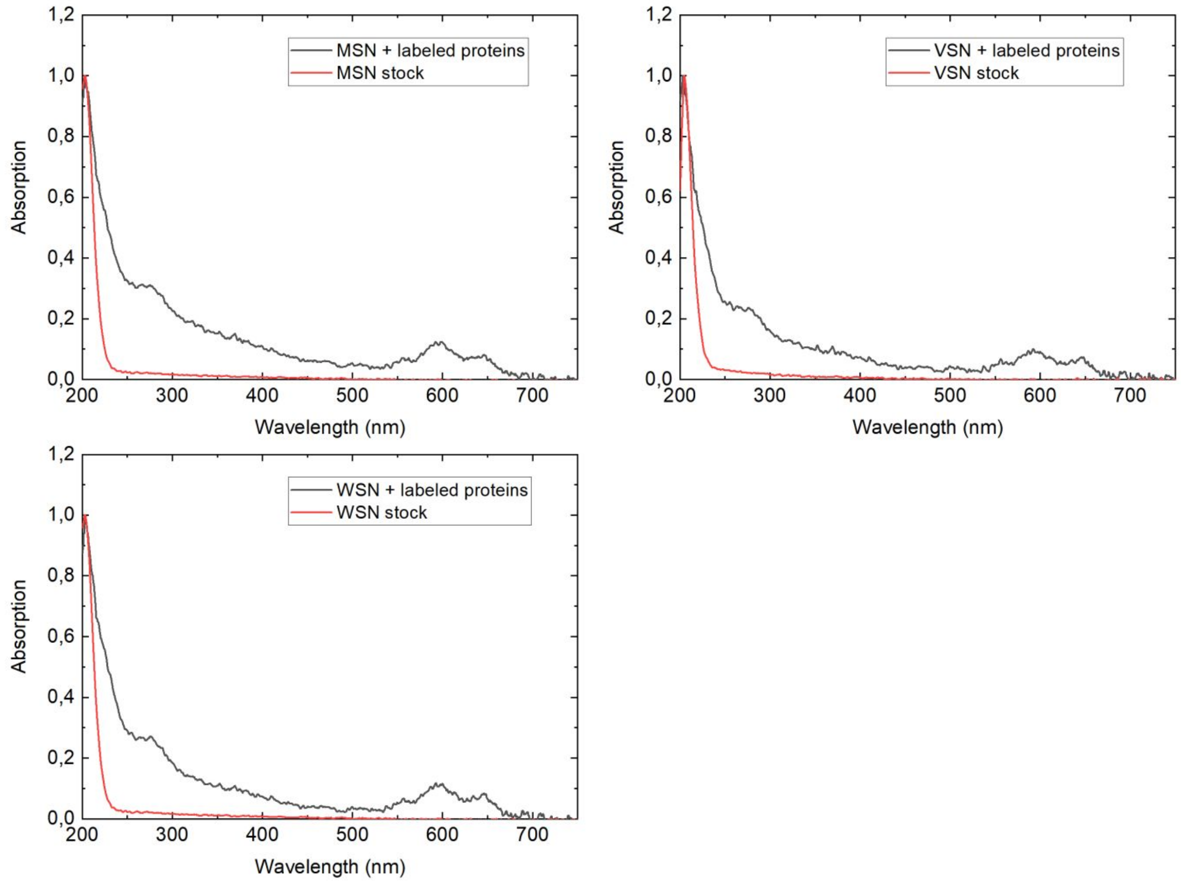

Figure S2 UV-Vis spectra of protein corona formed on silica nanoparticles with different surface morphology

Nanoparticle surface characterization

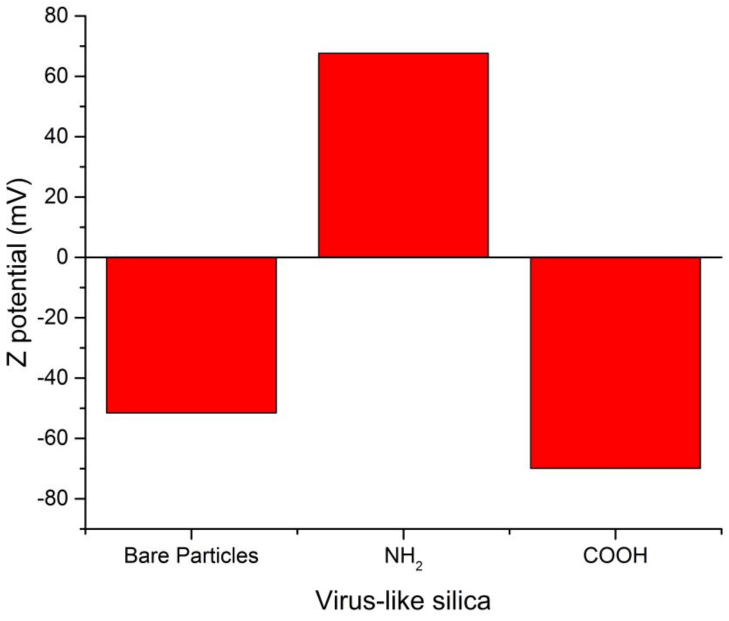

Figure S3 Zeta-potential change of VSN in different steps throughout the surface functionalization. Zeta-potentials of VSN particles change due to charges on the functionalized group, indicating successful surface modification.

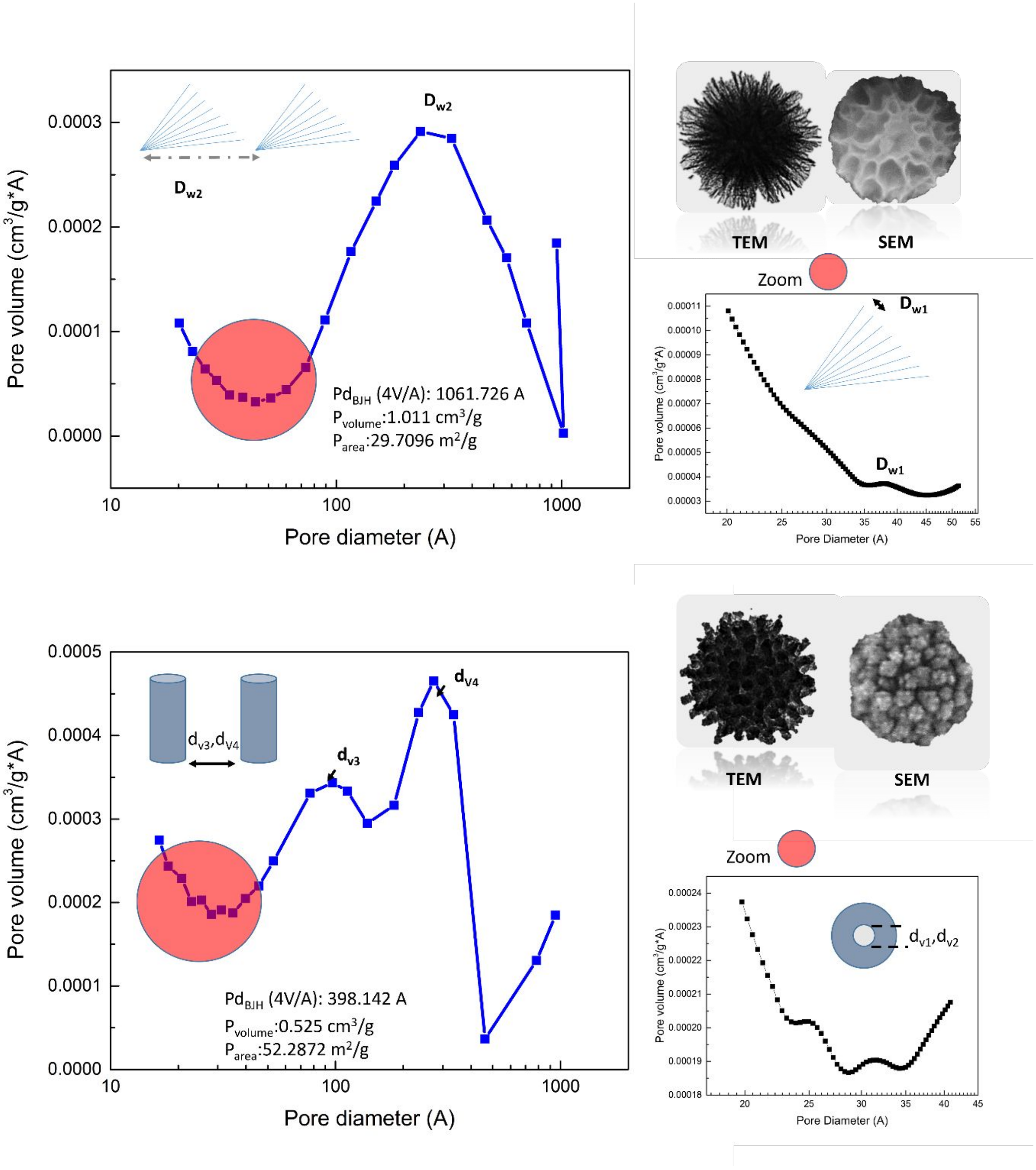

Figure S4 BET results of WSN (top) and VSN (bottom) nanoparticles. In the top WSN figure, D_{w1} and D_{w2} represent intra- and inter-wrinkle distances respectively, and in the bottom VSN figure, d_{v1}, d_{v2} represent bimodal inner tube diameters while d_{v3}, d_{v3} are bimodal inter-tube distances. The pore size and specific surface area of the MSN were obtained by physical adsorption of gases N 2 at 77 K using Micromeritics TriStar 3000 V6.04 A .The sample was outgassed at 100 ° C for 4 h prior to the adsorption measurements. The specific surface area (SBET, m 2 /g) was determined by multipoint Brunauer-Emmett-Teller (BET) method in the region of the isotherm, which is limited by the range of relative pressure P/P0 = 0.049–0.25. The pore size distribution is calculated from adsoption/desorption isotherms by the Barrett-Joyner-Halenda (BJH) method.

Cross-talk correction

Fluorescence cross-talk happens in our measurement due to the excitation and emission collection of at most two dyes at the same time. To correct for this, we alternately measured confocal and STED signals of particles labeled with only one dye using identical imaging parameters in real multicolor STED measurement, calculated the cross-talk factors by mean fluorescence intensities of nanoparticles. The measured cross-talk factors σ_{ct} is summarized in Table 4.

Table 4 measured cross-talk factors

	Ch1 (Red)	Ch2 (Orange)	Ch3 (Blue)
STAR Red	1	0.38%	0.55%
STAR Orange	11%	1	18%
Chromeo 494	11%	25%	1

In a multicolor STED measurement, we define measured STED intensity in Star Red, Star Orange and Chromeo 494 channels as I_R, I_o and I_b, and corrected values I_R^*, I_o^*, and I_b^*, therefore:

$$\begin{bmatrix} I_R^* \\ I_o^* \\ I_b^* \end{bmatrix} = \begin{bmatrix} 1 & A_1 & B_1 \\ A_2 & 1 & B_2 \\ A_3 & B_3 & 1 \end{bmatrix}^{-1} \times \begin{bmatrix} I_R \\ I_o \\ I_b \end{bmatrix}, \tag{1}$$

which can be written as $F_{cor} = \sigma_{ct}^{-1} F_{meas}$, where $F_{cor} = \begin{bmatrix} I_R^* \\ I_o^* \\ I_b^* \end{bmatrix}$, and $F_{meas} = \begin{bmatrix} I_R \\ I_o \\ I_b \end{bmatrix}$.

Line profiles of protein corona on single nanoparticles internalized by cells

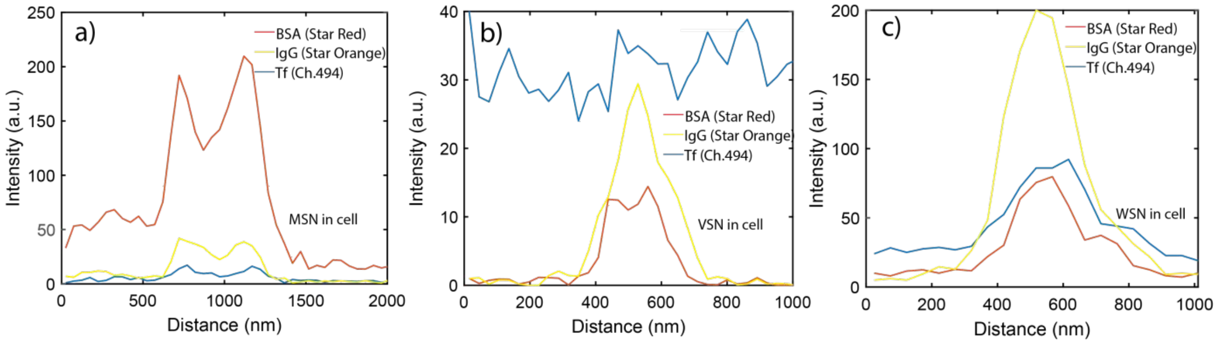

Figure S5 a) – c) Multicolor STED line profiles of protein corona on single MSN, VSN and WSN nanoparticles respectively.

Matlab codes for STED analysis

Matlab codes accompanying this manuscript has been made publicly available free of charge under GPL-3.0 license, and can be downloaded at: https://github.com/yetiswang/STED_analysis. The attached Matlab codes were used to perform all data analysis as demonstrated in our manuscript.
